# Presenilin E318G variant and Alzheimer’s disease risk: the Cache County study

**DOI:** 10.1186/s12864-016-2786-z

**Published:** 2016-06-29

**Authors:** Ariel A. Hippen, Mark T. W. Ebbert, Maria C. Norton, JoAnn T. Tschanz, Ronald G. Munger, Christopher D. Corcoran, John S. K. Kauwe

**Affiliations:** Department of Biology, Brigham Young University, Provo, UT USA; Department of Psychology, Utah State University, Logan, UT USA; Department of Nutrition, Dietetics, and Food Science, Utah State University, Logan, UT USA; Department of Mathematics and Statistics, Utah State University, Logan, UT USA

**Keywords:** Alzheimer’s disease, Presenilin, Rare variants, Epistasis

## Abstract

**Background:**

Alzheimer's disease is the leading cause of dementia in the elderly and the third most common cause of death in the United States. A vast number of genes regulate Alzheimer’s disease, including Presenilin 1 (*PSEN1*). Multiple studies have attempted to locate novel variants in the *PSEN1* gene that affect Alzheimer's disease status. A recent study suggested that one of these variants, *PSEN1 E318G* (rs17125721), significantly affects Alzheimer's disease status in a large case–control dataset, particularly in connection with the *APOE*ε*4* allele.

**Methods:**

Our study looks at the same variant in the Cache County Study on Memory and Aging, a large population-based dataset. We tested for association between *E318G* genotype and Alzheimer’s disease status by running a series of Fisher’s exact tests. We also performed logistic regression to test for an additive effect of *E318G* genotype on Alzheimer’s disease status and for the existence of an interaction between *E318G* and *APOE*ε*4*.

**Results:**

In our Fisher’s exact test, it appeared that *APOE*ε*4* carriers with an *E318G* allele have slightly higher risk for AD than those without the allele (3.3 vs. 3.8); however, the 95 % confidence intervals of those estimates overlapped completely, indicating non-significance. Our logistic regression model found a positive but non-significant main effect for *E318G* (*p* = 0.895). The interaction term between *E318G* and *APOE*ε*4* was also non-significant (*p* = 0.689).

**Conclusions:**

Our findings do not provide significant support for *E318G* as a risk factor for AD in *APOE*ε*4* carriers. Our calculations indicated that the overall sample used in the logistic regression models was adequately powered to detect the sort of effect sizes observed previously. However, the power analyses of our Fisher’s exact tests indicate that our partitioned data was underpowered, particularly in regards to the low number of *E318G* carriers, both AD cases and controls, in the Cache county dataset. Thus, the differences in types of datasets used may help to explain the difference in effect magnitudes seen. Analyses in additional case–control datasets will be required to understand fully the effect of *E318G* on Alzheimer's disease status.

## Background

Alzheimer’s disease (AD) is the most common type of dementia in the elderly and the third most common cause of death in the United States [1]. It is notoriously difficult to diagnose, with highly variable clinical symptoms and no existing laboratory test able to detect it conclusively [2]. The two primary markers of AD, amyloid plaques and neurofibrillary tangles, can usually only be detected at autopsy.

AD’s clinical complexity is compounded by its complex etiology, and a vast number of genes have been implicated [3, 4]. Ridge et. al. recently estimated that 33% of AD phenotypic variation can be explained by genetic mutations, and suggested that 25% of the total variation may be explained by currently-unknown rare mutations [5]. Ebbert et. al. further demonstrated that epistasis plays a critical role in Alzheimer’s and may explain a significant proportion of the missing heritability [6]. The Presenilin 1 gene (*PSEN1*) is one of the genes known to affect Alzheimer’s disease risk [7]; it codes for a transmembrane protein that forms the core of the gamma secretase complex, an enzyme that cleaves amyloid precursor protein into β-amyloid peptides [8]. *PSEN1* mutations have been shown to affect amyloid precursor protein cleavage, resulting in amyloid β-42 peptides. These longer, more hydrophobic fragments tend to form into plaques that are associated with damaged brain tissue, making amyloid β-42 levels an important pathological marker for AD [9].

Another relevant biomarker in pre-symptomatic Alzheimer’s is the tau protein, which is responsible for stabilizing microtubules in the brain. However, phosphorylated tau (ptau) is less able to perform this function, allowing the microtubules to become disorganized and form neurofibrillary tangles [10]. An increased phosphorylation of tau has been associated with increased amyloid deposition into plaques [11]. Fagan et. al. observed that the ratio of amyloid β-42 to ptau is associated with cognitive decline [12] and thus the two combined could make a probable biomarker for preclinical Alzheimer’s disease.

Multiple studies have identified novel variants in the *PSEN1* gene that affect Alzheimer’s disease status [13–15]. A recent study by Benitez et. al. found that one such mutation, *PSEN1 E318G* (rs17125721), increases the ratio of tau to Aβ-42 and ptau to Aβ-42 in the cerebrospinal fluid; as discussed previously, these ratios can be an important indicator of a preclinical AD phenotype [16]. The Benitez et. al. study also suggests a potential gene-gene interaction between *PSEN1* and *APOE*. Specifically, it suggests that *E318G* carriers who are also heterozygous carriers of the *APOE*ε*4* allele have a similar AD risk as individuals who are homozygous for *APOE*ε*4* and twice the risk of those heterozygous for *APOE*ε*4* without the *E318G* allele.

Gene-gene interactions are an important element of AD etiology [17–20], and an interaction between *PSEN1 E318G* and *APOE* could have an important function in the genetic pathways of the disease, but the existence of such an effect has not yet been validated in other datasets. Here, we attempt to replicate the effects of *E318G* in connection to *APOE* on Alzheimer’s status in a population-based dataset.

## Methods

Samples for this study came from the Cache County Study on Memory Health and Aging. When the study began in 1994, the 5092 subjects therein represented approximately 90% of all residents age 65 and older in Cache County, Utah. Case–control status was determined through a series of clinical dementia evaluations and cognitive assessments, including the Modified Mini-Mental State Exam-Revised [21]. These assessments were administered every three years over a period of twelve years. Cases were defined as those subjects meeting criteria determined by the National Institute of Neurological Disorders and Stroke–Alzheimer’s disease and Related Disorders Association to indicate possible or probable AD. Individuals with dementia only showed symptoms of late-onset AD with no other comorbid forms of dementia. Cognitively normal individuals were free of any symptoms of AD and other comorbid forms of dementia at all stages of screening. There were no early-onset Alzheimer’s cases in the dataset. Additional information about data collection and phenotype determination has been reported previously [22].

We genotyped the *PSEN1 E318G* locus (rs17125721) for 3420 individuals using a custom TaqMan assay. Genotypes were called based on cluster analyses using the default settings in the TaqMan genotyper software (Thermo Fisher). The marker had a call rate of greater than 98%. Of the 3420 individuals, 478 were clinically ascertained Alzheimer’s disease cases and 2942 were cognitively normal controls.

We excluded all individuals for whom *APOE* and *E318G* genotyping or Alzheimer's disease case/control status were unavailable. We used Fisher’s exact test to calculate odds ratios for effect of *APOE*ε*4* status on AD risk in *E318G* carriers and *E318G* non-carriers. To determine if the effect of *E318G* on AD risk was different for *APOE*ε*4* homozygotes versus heterozygotes we calculated the odds ratios for each combination of *E318G* carrier/non-carrier status and their number of *APOE*ε*4* alleles (0, 1 or 2).

We used the odds ratios described above to calculate the synergy factor between *E318G* status and *APOE*ε*4* status. Synergy factors are the ratio of the observed and expected odds ratios for the two interacting SNPs. Assuming there is no synergy between the SNPs (i.e., the SNPs are independent); the expected odds ratio then equals the product of the individual odds ratios. The synergy factor is calculated by dividing the observed odds ratio by the expected odds ratio for the interacting SNPs. A synergy factor that deviates from 1 suggests a statistical interaction between the SNPs [19],[20]. We calculated the synergy factor of *E318G* and *APOE*ε*4* to test further the existence of an interaction between these SNPs and their effect on Alzheimer’s disease risk.

We ran a logistic regression model using case/control status as the response, with *E318G*, age, gender, and *APOE*ε*4* positive or negative status as covariates. We also included an interaction between *APOE* and *E318G* to test the finding by Benitez et. al. that carriers of both *E318G* and *APOE*ε*4* were more at risk of developing AD than carriers of either mutation alone. We then tested the *E318G* mutation's effect on AD status independent of *APOE*ε*4* in a second logistic regression model by using only *APOE*ε*3* homozygotes in the Cache County dataset (*n* = 1916). In this model, we included *E318G*, age, and gender as covariates.

To determine if we had sufficient statistical power to observe an effect of *E318G* on AD status in connection to *APOE*ε*4*, we calculated the minimum discernible effect size of *E318G* in all individuals in the Cache County Study using a power analysis [23]. We used sample size, *E318G* exposure, and AD status to find the smallest effect size we could detect with 80% power. If we had sufficient power to detect the kind of odds ratios that were found by Benitez et. al., then we could be confident that our sample size was large enough to discover the same effect if it existed in the Cache County dataset. Then, to determine if we had enough power to see an effect of *E318G* on AD status independent of *APOE*ε*4*, we calculated the minimum discernible effect size of *E318G* in *APOE*ε*3* homozygotes.

## Results

It would appear that *APOE*ε*4* carriers with an *E318G* allele have slightly higher risk for AD than those without the allele (3.3 vs. 3.8); however, the 95 % confidence intervals of these two estimates overlap completely (Table 1). Similarly, comparisons of odds ratios for AD risk based on *E318G* and number of *APOE*ε*4* alleles revealed that *E318G* carriers with one *APOE*ε*4* allele were at a higher—but non-significant—risk for AD (OR = 3.5, CI = 1.0-12.7, *p* = 0.043) compared to *APOE*ε*4* heterozygotes who did not carry the *E318G* allele (OR = 3.0, CI = 2.4 - 3.7, *p* < 2.2 × 10^−16^). *E318G* carriers with two *APOE*ε*4* alleles had a larger odds ratio (OR = 9.1, CI = 0.1 - 759.3, *p* = 0.197) than non-carriers (OR = 7.5, CI = 4.6-12.0, *p* = 2.4 × 10–15), but the confidence interval was extremely large and the result was not significant (Table 2). Based on our synergy factor calculation, the odds ratios obtained from the Fisher's exact test comparing Alzheimer’s disease risk based on *APOE*ε*4* presence or absence did not give evidence of a significant interaction between *E318G* and *APOE*ε*4* (Synergy factor = 1.17, *p* = 0.78).

**Table 1:** Results from Fisher’s exact test. The odds ratio for Alzheimer’s Disease risk for individuals who were carriers of E318G and APOEε4 was higher than the odds ratio for those who only carried APOEε4, but the confidence intervals overlap

Alzheimer’s risk based on E318G status and APOEε4 Status						
Strata		Cases	Controls	*P* value	OR (95 % CI)	Power
E318G +	APOEε4 (+)	8	20	0.0172	3.8 (1.1–13.3)	0.66
	APOEε4 (−)	8	77			
E318G –	APOEε4 (+)	253	765	<2.2 × 10^−16^	3.3 (2.7–4.0)	1.0
	APOEε4 (−)	209	2080

**Table 2:** Results from Fisher’s exact test. The odds ratios observed were higher for E318G+ individuals than E318G– individuals with the same number of APOEε4 alleles, but the 95 % confidence intervals for these groups overlap

Alzheimer’s risk based on E318G status and number of APOEε4 Alleles						
Strata		Cases	Controls	*P* value	OR (95 % CI)	Power
E318G +	APOEε44	1	1	0.197	9.1 (0.1–759.3)	0.04
	APOEε4 (+)	7	19	0.043	3.5 (1.0–12.7)	0.16
	APOEε4 (−)	8	77			
E318G –	APOEε44	36	48	2.4 × 10^−15^	7.5 (4.6–12.0)	1.0
	APOEε4 (+)	217	717	<2.2 × 10^−16^	3.0 (2.4–3.7)	1.0
	APOEε4 (−)	209	2080			

In our first logistic regression model, which considered all individuals in the dataset and included an interaction term between *APOE*ε*4* and *E318G*, we found a positive but non-significant main effect for *E318G* (*p* = 0.895). The interaction term between *E318G* and *APOE*ε*4* was also non-significant (*p* = 0.689). The second logistic regression model, which included only those who were homozygous for the non-disease-causing *APOE*ε*3* allele, also did not have a significant additive effect for *E318G* (*p* = 0.826). Our power analysis indicated that, with a sample size of 3420, we had 80% power to detect an odds ratio of 1.9 or bigger if one existed (alpha = 0.05). For the second logistic regression model, with a sample size of 1916 APOEε3 heterozygotes, we had 80% power to detect an odds ratio of 2.4 or larger (alpha = 0.05).

## Discussion

We failed to detect significant associations with *E318G* and AD as described by Benitez et al. While the direction of our results are consistent with those reported by Benitez et al., our results provide little support that the *E318G* allele increases risk for AD in *APOE*ε*4* carriers (Tables 1 and 2). Further studies will be necessary to clarify the effects of *E318G* on AD. The lack of replication in rare variant associations and with interactions can be difficult to interpret, as these associations are particularly sensitive to genetic and phenotypic heterogeneity. While our calculations indicate the overall sample is adequately powered to detect the sort of effect sizes observed by Benitez et al., the power analyses of our Fisher’s exact tests indicate that our study of the partitioned data was underpowered, particularly in regards to the low number of *E318G* carriers, both AD cases and controls, in the Cache county dataset. Differences in ascertainment and population may have presented challenges for replication in the Cache County Study of the finding of Benitez et al. As such, we believe that further analyses using samples that are more similar to those used in the original report will be necessary to determine the significance of the interaction between the *APOE*ε*4* allele and *E318G* on Alzheimer's disease risk.

## Conclusions

Rare variants play a significant role in Alzheimer’s disease phenotypic variance. The number of possible variants with a functional effect, as well as the existence of epistatic interactions, means that many further studies will be required to fully characterize rare variants’ roles. Our study did not find any evidence of *PSEN1 E318G* having a significant effect on Alzheimer’s disease status, but it is possible that significance could be found using larger, case–control datasets. More studies involving greater number of rare variants, examined both independently and in combination, will help to make the genetic etiology of Alzheimer’s disease clearer and more clinically actionable.
